# The European Drug–Drug Interaction (EuroDDI) Study Protocol: A Cross‐Country Comparison of Drug–Drug Interaction Prevalence in the Older Community‐Dwelling Population

**DOI:** 10.1002/pds.70092

**Published:** 2025-01-07

**Authors:** John E. Hughes, Enrica Menditto, Sara Mucherino, Valentina Orlando, Aida Moreno‐Juste, Antonio Gimeno‐Miguel, Beatriz Poblador‐Plou, Mercedes Aza‐Pascual‐Salcedo, Francisca González‐Rubio, Ignatios Ioakeim‐Skoufa, Kathleen Bennett, Caitriona Cahir

**Affiliations:** ^1^ School of Population Health RCSI University of Medicine and Health Sciences Dublin 2 Ireland; ^2^ CIRFF, Center of Pharmacoeconomics and Drug Utilization Research, Department of Pharmacy University of Naples Federico II Naples Italy; ^3^ EpiChron Research Group, Aragon Health Sciences Institute (IACS), IIS Aragón Miguel Servet University Hospital Zaragoza Spain; ^4^ Network for Research on Chronicity, Primary Care, and Health Promotion (RICAPPS) Institute of Health Carlos III (ISCIII) Madrid Spain; ^5^ San Pablo Primary Care Health Centre Aragon Health Service (SALUD) Zaragoza Spain; ^6^ Drug Utilization Work Group Spanish Society of Family and Community Medicine (semFYC) Barcelona Spain; ^7^ Department of Drug Statistics, Division of Health Data and Digitalisation Norwegian Institute of Public Health Oslo Norway; ^8^ Emerging Technologies Advisory Group ISACA Schaumburg Illinois USA

**Keywords:** adverse drug reactions, cardiovascular, common data model, drug interactions, elderly, medication safety, nervous system, pharmacoepidemiology, polypharmacy, protocol

## Abstract

**Background:**

Drug–drug interactions (DDIs), highly prevalent amongst the elderly, can lead to avoidable medication‐related harm. Cardiovascular and central nervous system (CNS) drugs are commonly implicated. To date, there is no consensus on how to measure DDIs, making comparisons across countries challenging.

**Objective:**

To (i) establish a common data model (CDM) to measure DDI prevalence in the older (aged ≥ 70 years) community‐dwelling population of three European countries and (ii) compare and describe cardiovascular and CNS DDI prevalence rates across these countries.

**Methods:**

This cross‐country study will apply a harmonised method of DDI identification and analysis using the WHO ATC classification system and national pharmacy claims data from three European countries (Ireland, Italy, Spain). Patients aged ≥ 70 years dispensed ≥ 2 medications during 2016 will be identified from each country's national database. ‘Severe’ cardiovascular and CNS DDIs (i.e., may result in a life‐threatening event/permanent detrimental effect) will be identified using the British National Formulary and Stockley's Drug Interactions. Two separate lists of ‘severe’ DDIs, per medications reimbursed, will be applied to each database: (i) DDIs relevant to each individual country and (ii) DDIs relevant to all three countries. DDIs will be defined as co‐dispensed (same day) and concomitantly (±7 days) dispensed.

**Results:**

Descriptive statistics, including DDI prevalence and 95% confidence intervals, will be reported for each country. Prevalence will be pooled and compared across countries using random effects models and meta‐regression, where feasible.

**Conclusion:**

The EuroDDI study will develop a harmonised method to measure and compare DDI prevalence across health‐related databases in Europe.

**Summary**

- Drug–drug interactions are an avoidable cause of medication‐related harm in the older population; yet prevalence estimates are poorly defined due to lack of standardised methods with robust validity.
- We are a European collaborative research group that will use real‐world data to measure ‘severe’ cardiovascular and central nervous system drug–drug interaction prevalence in the older community‐dwelling population using a common data model.
- Findings will inform public health policy and drug–drug interaction health outcomes research at local and national European level.

## 1 Background

A drug–drug interaction (DDI) occurs when the effect of one drug is altered by the use of another drug [1]. In general, DDIs are considered to be a predictable cause of medication‐related harm [2]. They can result in adverse health outcomes [3, 4, 5, 6, 7]; increased costs to healthcare systems [8, 9]; and, in some instances, have been associated with mortality [10]. The older population is at greatest risk, primarily due to the rising burden of chronic disease and associated polypharmacy, defined as the regular use of five or more medications, as well as age‐related physiological decline [11, 12]. Indeed, while the burden of polypharmacy in the older population is well‐described in the literature [13, 14, 15, 16], trends in DDI prevalence, particularly across Europe, are less well‐understood, and meaningful comparison of DDI prevalence across populations is often challenging, if not impossible [17].

In the past decade, many studies have investigated DDI prevalence in the older community‐dwelling population, with most measuring the prevalence of potentially clinically important DDIs [17]. In Europe, prevalence estimates range from 0.8% to 54.3% [18, 19, 20, 21, 22, 23, 24, 25], and in Australia, approximately 1.5% of the older population is potentially exposed [26], while in the United States prevalence estimates range from 7.7% to 30.2% [27, 28, 29]. The variation in estimates may be due to differences in sample sizes or, more broadly, clinical differences across these populations, such as the rate of disease and medication burden; however, differences in the method used to identify DDIs also contribute to this variation [17]. A recent systematic review and meta‐analysis of 31 studies involving more than 17 million community‐dwelling individuals aged ≥ 65 years reports a pooled DDI prevalence of 28.8% (95% CI 19.3, 40.7), with significant heterogeneity (*p* < 0.10; I2 = 100%; tau2 = 2.13) largely explained by the different DDI identification methods used across studies [17]. In order to facilitate meaningful measurement and comparison of DDI prevalence across different older populations and countries, a standardised (harmonised) methodology is needed. However, to date, there is no consensus on how to measure DDIs, making comparisons across countries challenging.

### 1.1 Aims and Objectives

The EuroDDI study aims to develop and implement a common data model (CDM) to measure DDI prevalence across different populations and countries in Europe. The CDM will be tested in three different European cohorts to identify DDIs involving pharmacological agents used to treat conditions of the cardiovascular and central nervous system (CNS). These two physiological systems are consistently shown to have the highest prevalence of potential DDIs in the older population [22] and are commonly implicated in adverse outcomes [7, 30]. The specific objectives are as follows:

1. to establish the European drug–drug interaction common data model (EuroDDI‐CDM);
2. to assess the feasibility of a collaborative cross‐country comparison of DDI prevalence using a harmonised DDI identification methodology and based on pooled primary care dispensing data in older populations (aged ≥ 70 years) in three European countries; and
3. to establish and compare cardiovascular and CNS DDI prevalence rates and to identify the most frequently dispensed DDIs in older community‐dwelling populations across the three different European cohorts.

## 2 Methods

This protocol has been developed in accordance with International Society of Pharmacoepidemiology Guidelines for Good Pharmacoepidemiology Practice [31].

### 2.1 Design

Cross‐sectional cohort.

### 2.2 Data Sources

Three European pharmacy claims databases will be used: (1) the Health Services Executive Primary Care Reimbursement Services General Medical Services (HSE‐PCRS GMS) database with linkage to The Irish LongituDinal study on Ageing (TILDA), Ireland; (2) the CaReDB data warehouse from Campania Region, Italy; and (3) the EpiChron Cohort in Aragon, Spain. Each database collects data on drugs dispensed from pharmacies in the community/primary care setting and has been validated for pharmacoepidemiology research [32, 33, 34]. Within each database, the reimbursement claim date for drug dispensation is available, and drugs are classified using the World Health Organization (WHO) Anatomical Therapeutic Chemical (ATC) classification system, which allows for direct comparison between countries [35]. Characteristics and common elements of the three databases are summarised in Table 1. Further detail on each database is provided below. A common observation time period (January 1, 2016 to December 31, 2016) will be used.

**TABLE 1:** Characteristics of the healthcare systems in the EuroDDI countries.

	Ireland	Italy	Spain^a^
Database	TILDA linked to HSE‐PCRS‐GMS	CaReDB data warehouse from Campania	EpiChron
Observation period	01/01/2016–31/12/2016	01/01/2016–31/12/2016	01/01/2016–31/12/2016
Denominator	Number of individuals dispensed ≥ 2 drugs (distinct ATC codes) on any pharmacy claim during the time period	Number of individuals dispensed ≥ 2 drugs (distinct ATC codes) on any pharmacy claim during the time period	Number of individuals dispensed ≥ 2 drugs (distinct ATC codes) on any pharmacy claim during the time period
Health care system	Predominantly tax‐funded; two‐tiered public and private system	Universal	Universal
Physicians/100 000 inhabitants^b^	319	405	725
Unique Health Identifier	GMS medical card number	TS medical card number	AR medical card number
Date of claim	Yes	Yes	Yes
Quantity dispensed	Yes	Yes	Yes

^a^ Aragon.

^b^ EuroSTAT 2016.

### 2.3 Study Population and Pharmacy Claims Databases

- IRELAND.

#### 2.3.1 Health Service Executive‐Primary Care Reimbursement Service General Medical Services (HSE‐PCRS GMS)

The HSE‐PCRS GMS scheme is the largest pharmacy claims database in Ireland, covering more than 40% of the general Irish population. It is means tested and provides free health services, including subsidised medications, to eligible persons in Ireland. Eligibility for the GMS scheme (sometimes referred to as the ‘medical card scheme’) is on the basis of income related means‐testing. Automatic entitlement for those aged ≥ 70 years occurred between July 2001 and December 2008; however, since January 2009, means‐testing was introduced, but with a higher income threshold than the general population. As of 2013, 90% of men and 94% of women in the general population aged ≥ 70 years were eligible, therefore, representing a unique population‐based resource [36]. Within the HSE‐PCRS GMS pharmacy claims database, drugs dispensed are coded using the World Health Organization (WHO) Anatomical Therapeutic Chemical (ATC) classification system and defined daily doses, strength, quantity and route of administration for each drug dispensed are all available. At each wave of data collection, TILDA participants with GMS eligibility are asked to provide consent to link their pharmacy claims data to study data. Pharmacy claims data will be extracted for TILDA participants aged ≥ 70 years with GMS eligibility for the wave‐4 (01/01/2016 to 31/12/2016) period. To be included in this study, participants will be required to have at least two medicines (distinct ATC codes) on any pharmacy claim during the time period. Ethical approval for TILDA was granted by the Faculty of Health Sciences Ethics Committee, Trinity College Dublin.

#### 2.3.2 The Irish LongituDinal Study on Ageing (TILDA)

TILDA is a nationally representative prospective cohort study of community‐dwelling individuals aged ≥ 50 years in Ireland. The first wave of data collection began in October 2009 through to February 2011 (*N* = 8175 participants aged ≥ 50 years), where participants completed a computer‐aided personal interview (CAPI) and a health assessment measuring their health, economic and social circumstances. Data collection waves occur once every 2 years; to date, six waves of data have been collected. Further information on TILDA's study design and sampling framework has previously been described [37]. Linked TILDA‐PCRS data will be used for the proposed research. Wave‐4 (2016) of TILDA includes approximately *n* = 1200 participants aged ≥ 70 years with linked medical card pharmacy claims data.

Ethical approval was obtained from the RCSI research ethics committee (REC #REC202209011).

ii ITALY.

#### 2.3.3 The Campania Region Database (CaReDB)

The Campania Region Database (CaReDB) is a health‐related data warehouse containing data collected during the monitoring of administrative health databases. These databases are connected to each other through a record‐linkage system that uses as a key the identification code of the patient properly encrypted in accordance with privacy regulations. Data of CaReDB contain information on pharmaceutical prescriptions and hospital discharge forms of all subjects living in Campania, Southern Italian region of about 6 million inhabitants (10% of the national population). CaReDB is complete and includes data that have been validated in previous drug utilisation studies [33, 38, 39, 40, 41, 42, 43, 44, 45].

iii SPAIN.

#### 2.3.4 The EpiChron Cohort

The Spanish data used in this study are from the EpiChron Cohort, which was created in 2011 for the study of the epidemiology of chronic diseases and multimorbidity. This cohort is based on the demographic and clinical information of all public health care system users in the Spanish region of Aragon, which is linked at the patient level and then pseudonymised. This information is collected from patients' electronic health records from primary and hospital health care, pharmacy billing records and users' database. This open cohort is updated regularly; baseline data include information on 1 253 292 individuals of all ages (mean age 44.2 years, 50.5% women, 11.9% migrants, 37.5% multimorbid, mean burden of 1.7 chronic diseases and 4.3 drugs). A detailed description of the cohort profile regarding baseline information, data sources used and details on data curation and linkage procedures has previously been described [34]. This study has been favourably assessed by the Clinical Research Ethics Committee of Aragon (CEICA, PI20/646).

### 2.4 Data Extraction

**FIGURE 1:**
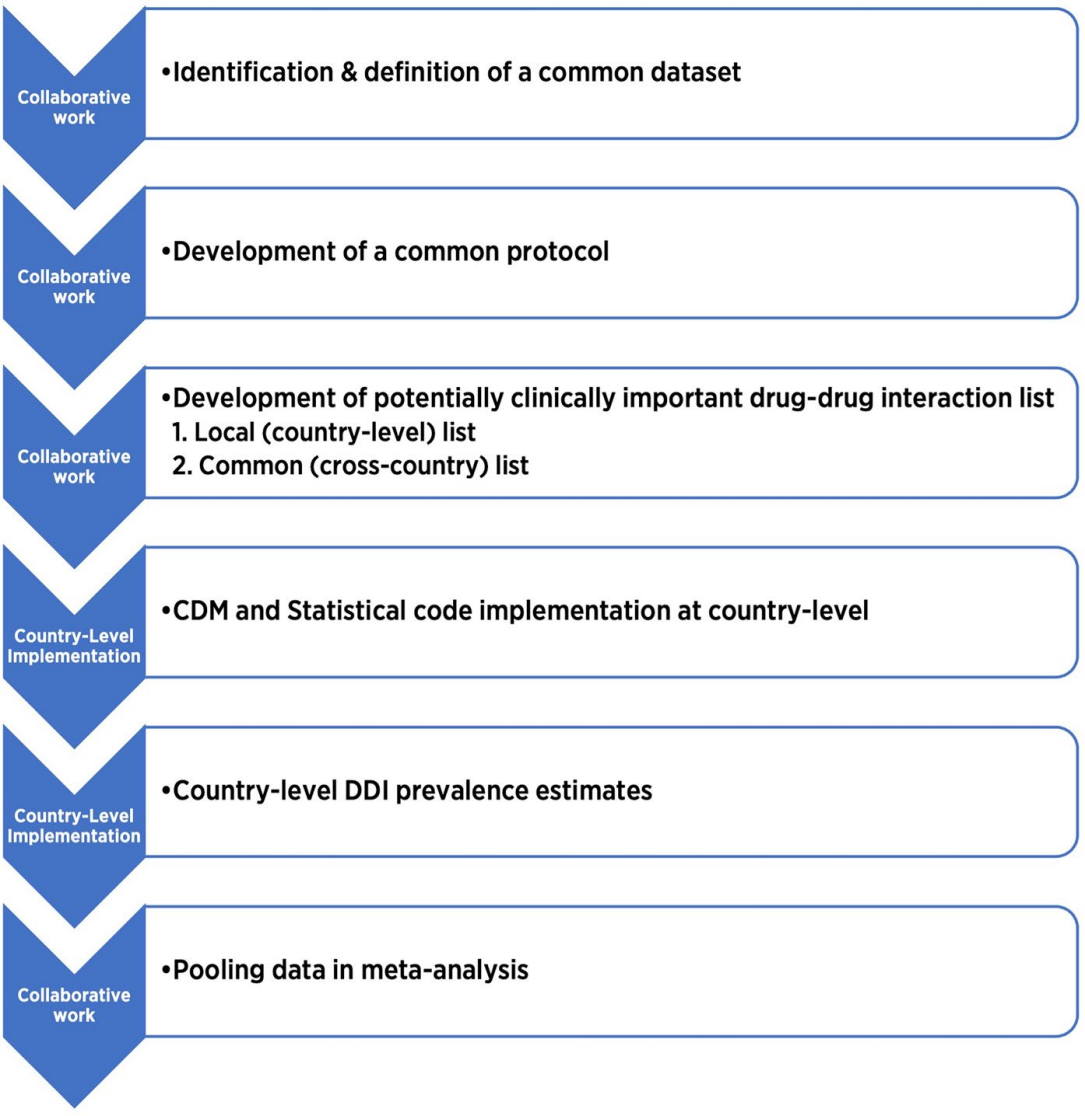
Six‐Step process used to implement the harmonised EuroDDI methodology. CDM, common data model; DDI, drug–drug interaction.

The harmonised EuroDDI prevalence methodology will be implemented using a 6‐step process (Figure 1). Data will be extracted locally by each country and transformed into a common data model (CDM). The CDM will include: sociodemographics (age, gender), chronic conditions and pharmacy claims data (ATC coded, date of pharmacy claim). A similar method has been used in a previous European collaborative study [43].

### 2.5 DDI Identification

**FIGURE 2:**
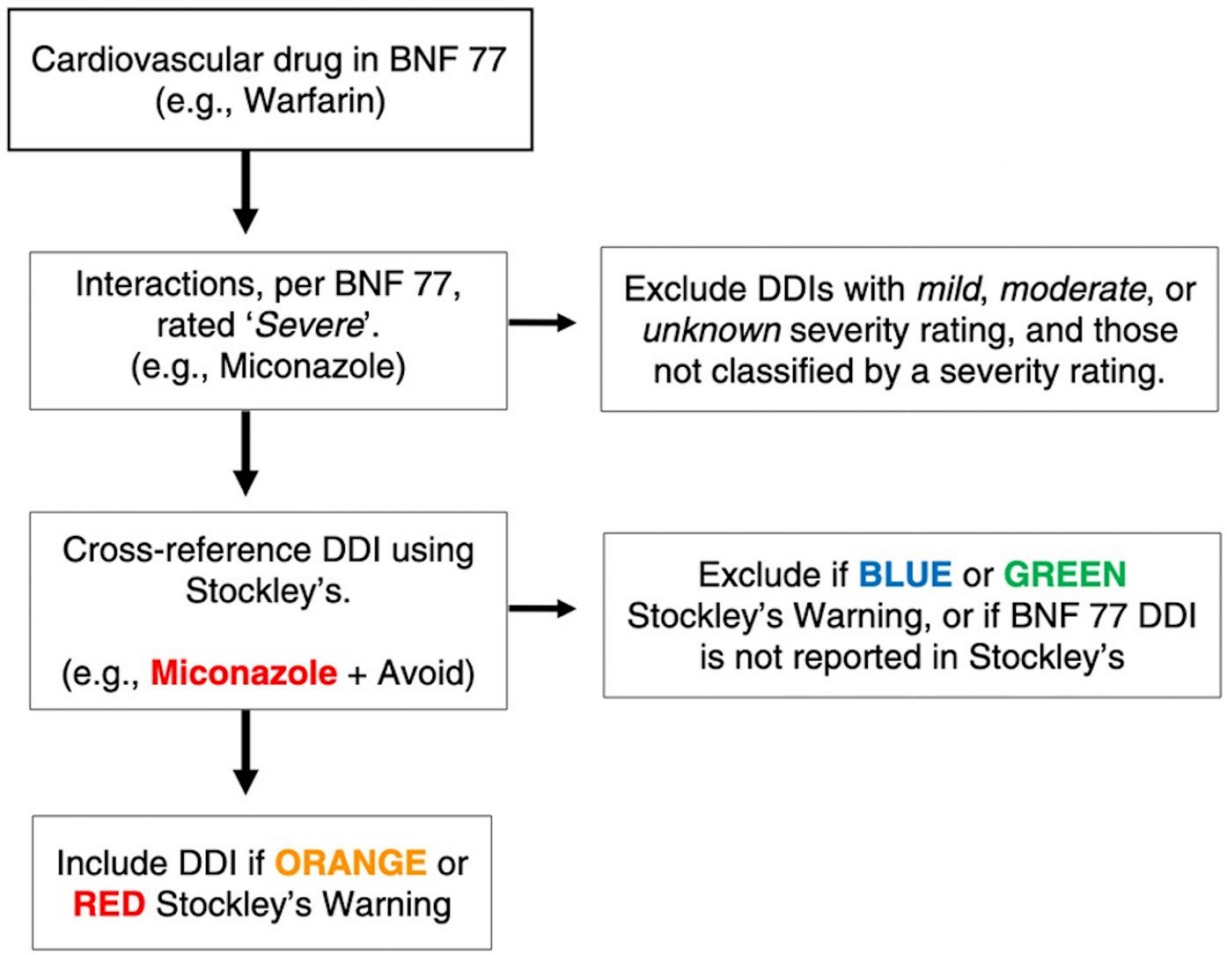
Identification of ‘Severe’ DDIs using BNF 77 and Stockley's Drug Interactions. Red DDIs: A life‐threatening or contraindicated combination; Orange DDIs: Dosage adjustment or close monitoring is needed; Blue DDIs: Give guidance about possible adverse effects and/or consider some monitoring; Green DDIs: No interactions or no interaction of clinical significance. BNF, British National Formulary; DDI drug–drug interaction.

We have previously developed a list of ‘severe’ potentially clinically important cardiovascular and CNS DDIs based on two pharmaceutical references commonly used in current clinical practice: the British National Formulary (BNF) and Stockley's Drug Interactions [25]. The BNF is commonly used in clinical practice in all three countries, and Stockley's Drug Interactions is considered to be the gold standard DDI reference [46]. This DDI list was developed by two pharmacists and contains approximately 5000 unique cardiovascular and CNS DDIs classified as being both ‘severe’ (i.e., the result may be a life‐threatening event or have a permanent detrimental effect) per the BNF 77 and also as being ‘a life‐threatening or contraindicated combination’ (red warning) or ‘dosage adjustment or close monitoring is needed’ (orange warning) per Stockley's Drug Interactions. Figure 2 summarises the methodology used to identify the ‘severe’ DDIs for analysis. A similar DDI identification method has been used in previous research [26].

### 2.6 Development of Country‐Specific DDI Lists

The interacting drug pairs contained within the list of potentially clinically important cardiovascular and CNS DDIs are coded using the World Health Organization (WHO) Anatomical Therapeutic Chemical (ATC) classification system [35]. In Europe, as in other parts of the Western world, medications are generally reimbursed based on the strength of evidence of benefit for the population, therapeutic value for money and fiscal capacity of individual member states [47]. Therefore, to reduce the risk of misclassification bias, for each European country, two separate potentially clinically important cardiovascular and CNS DDI lists will be used for analysis based on: (1) the medications reimbursed by each individual country and (2) medications that are commonly reimbursed by all three European countries.

### 2.7 Statistical Analysis

**FIGURE 3:**
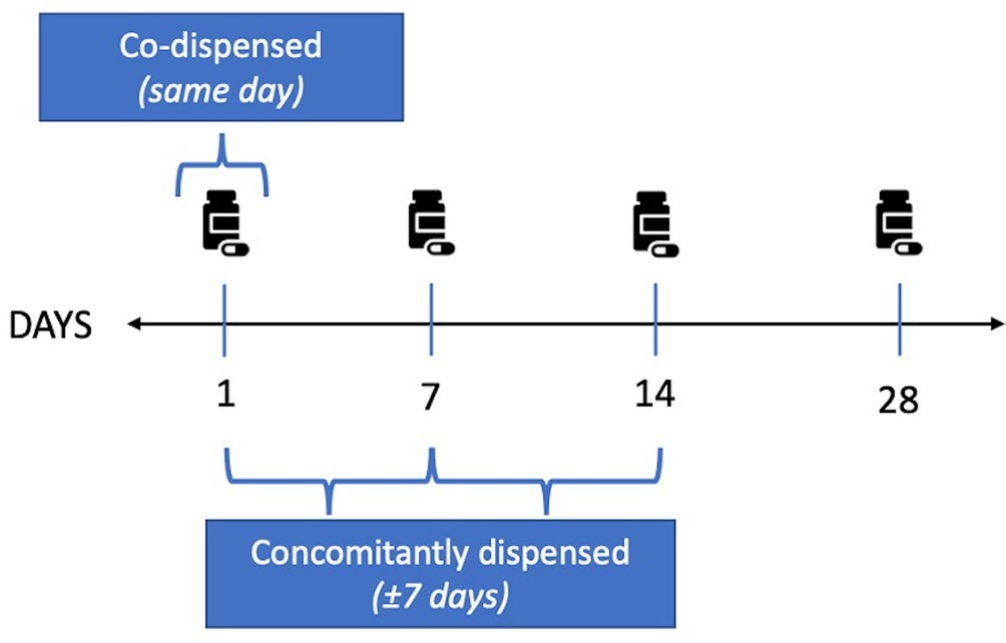
Drug dispensing patterns. Co‐dispensed (dispensed on the same day; for example amiodarone + warfarin [both dispensed on Day 1]); concomitantly dispensed (dispensed ± 7 days of date of claim; for example warfarin [day 7] + clarithromycin [day 14]).

To ensure a standardised application of the study methodology, a common statistical code will be developed for use across all three databases (countries). DDI prevalence and 95% confidence intervals (CI) will be reported per two different dispensing patterns (Figure 3): co‐dispensed (i.e., drug combinations dispensed on the same day) and concomitant (i.e., drug combinations dispensed within 7 days of each other) [48]. To understand the duration of potential DDI exposure, the number of acute (DDI occurred for a period of less than three consecutive monthly pharmacy claims) and chronic (the same DDI continued for three or more consecutive monthly pharmacy claims) DDIs will also be calculated per number of study participants for each country. At the individual country level, the prevalence of the most frequent DDIs per dispensing pattern (co‐dispensed, concomitantly dispensed, acute/chronic) and the potential effect and action required per the BNF 77 and Stockley's will be presented. DDIs which are common across all three countries will be identified and presented. The prevalence of Orange (*‘dosage adjustment or close monitoring is needed’*) and Red (*‘a life‐threatening or contraindicated combination’*) DDIs (per Stockley's) will also be reported. If sufficiently homogenous data are available, DDI prevalence will be pooled and compared across countries using random effects models and meta‐regression, where feasible.

## 3 Discussion

This protocol is for a European collaborative DDI study which will use a novel, evidence‐based methodology to identify potentially clinically important DDIs involving the cardiovascular system and CNS in the older community‐dwelling population in three European countries: Ireland, Italy and Spain. With the rising tide of polypharmacy in the older population [22], DDIs represent an important public health problem; not least because many DDIs are an often predictable cause of medication‐related harm [49]. In current clinical practice, DDI identification is generally reliant upon clinical decision support systems; however, these systems have limitations, including validity and the associated problem of ‘alert fatigue’ [50]. Identifying DDIs in real‐world data is therefore crucial in order to understand which DDIs should be routinely monitored and characterised for adverse health outcomes. Globally, as the world's older population continues to grow, there is a need to improve the measurement and monitoring of DDIs in order to mitigate the occurrence of avoidable patient harm. Implementing a CDM to analyse DDIs in different European health‐related databases will facilitate cross‐border comparative research, enhance patient safety and contribute to a better understanding of DDIs occurring in older populations and diverse healthcare settings. Findings from the EuroDDI project will have important implications for research, policy and clinical service. Specifically, estimating and comparing DDI prevalence in ageing populations across three European countries will be helpful to policy and decision makers when planning, developing and allocating healthcare resources to ensure ‘Medication Without Harm’ for the ageing population [51, 52]. To date, the lack of consensus on how to measure DDIs makes comparisons across different countries and populations challenging [17]. We believe the EuroDDI cross‐country collaborative project will overcome this challenge by implementing a harmonised methodology and CDM to measure meaningful DDI prevalence across three countries in Europe, which will permit plausible comparative analysis. Common DDIs, as well as differences in pharmacoepidemiology, will be reported; this will help to identify specific DDIs which should be the focus of future health outcomes research. This research will also inform European and country‐level pharmacovigilance activities to monitor and characterise the real‐world effect of these DDIs in older European populations. This protocol could be implemented in all countries where the ATC/DDD methodology is used, providing the opportunity for direct comparative DDI analysis.

### 3.1 Dissemination

Findings will be reported using the STrengthening the Reporting of Observational Studies in Epidemiology (STROBE) guidelines and the REporting of studies Conducted using Observational Routinely collected Data for pharmacoepidemiological research (RECORD‐PE) guidelines [53, 54]. Findings will be dissemination through poster and oral presentation at scientific meetings at national and international conferences and through peer‐reviewed journal publication.

### 3.2 Strengths and Limitations

This study will use objective real‐world pharmacy dispensing data derived from national population‐based pharmacy claims databases across three European countries. A novel, evidence‐based methodology will be used to identify potentially clinically important DDIs involving the cardiovascular and CNS in the older community‐dwelling population. The WHO ATC/DDD methodology will be used, which is the international standard for drug utilisation studies and permits plausible comparisons between countries. In addition, the use of a CDM will allow for direct comparison of DDI prevalence estimates across the older community‐dwelling populations of three European countries. This will facilitate the identification of DDIs which are common and which should be the focus of future health outcomes research. Given the inclusion of multiple European countries, the results may have broader applicability to other regions or countries with similar healthcare systems and demographics, increasing the generalisability of the findings. This research will use pharmacy refill claims data, and we assume that all medicines dispensed for a patient are taken. This may result in an over‐estimation of DDI prevalence, since patients may not take all medications dispensed. However, compared to self‐report or prescription data, pharmacy dispensing data are generally considered to be the gold standard of drug exposure information and have good validity for pharmacoepidemiology studies [55].

### 3.3 Plain Language Summary

Medicines are essential when it comes to treating illness and maintaining health. However, some medicine combinations do not mix well together and this can potentially cause medication‐related harm. Today, although it is common for older adults to use many medicines to maintain their health, this also means that they are at greatest risk of medication‐related harm. Currently, the number of older individuals receiving medication combinations which do not mix well together is poorly understood. It is also difficult to compare these numbers across countries, mainly due to the different methods currently used to identify and measure medications which do not mix well together. We are a group of European researchers from Ireland, Italy and Spain. We have developed a single list of medication combinations which do not mix well together, and we plan to use this to measure the number of older community‐dwelling individuals in all three countries exposed to these medication combinations. This will allow us to precisely compare numbers across countries and establish common medication combinations which can then be assessed for safety and public health interventions.

## Author Contributions

All authors were involved in the concept and design of the study. J.E.H. prepared the first draft of the protocol; C.C. and K.B. provided initial feedback; and all authors contributed subsequent feedback. All authors read and approved the final protocol.

## Conflicts of Interest

The authors declare no conflicts of interest.
